# A Highly Selective Fluorescent Probe for Hypochlorous Acid in Living Cells Based on a Naphthalene Derivative

**DOI:** 10.1155/2022/7649230

**Published:** 2022-02-14

**Authors:** Jingguo Sun, Junhong Xu, Qiujuan Ma, Guojiang Mao, Nannan Zhu, Meiju Tian, Linke Li, Shuzhen Liu

**Affiliations:** ^1^School of Pharmacy, Henan University of Chinese Medicine, Zhengzhou 450046, China; ^2^Department of Dynamical Engineering, North China University of Water Resources and Electric Power, Zhengzhou 450011, China; ^3^Henan Key Laboratory of Organic Functional Molecule and Drug Innovation, Collaborative Innovation Center of Henan Province for Green Manufacturing of Fine Chemicals, Key Laboratory of Green Chemical Media and Reactions, Ministry of Education, School of Chemistry and Chemical Engineering, Henan Normal University, Xinxiang 453007, China

## Abstract

Hypochlorous acid (HOCl) was crucial for maintaining the homeostasis in cells and plays vital roles in many physiological and pathological processes. In this work, a highly selective fluorescent probe for hypochlorous acid in living cells was constructed and prepared based on a naphthalene derivative. A naphthalene derivative was utilized as the fluorescent group, and *N*,*N*-dimethylthiocarbamate was applied as the selective recognition site for HOCl. Before adding HOCl, the fluorescent probe exhibited weak fluorescence. Upon adding HOCl, the fluorescent probe displayed remarkable fluorescence enhancement. The fluorescence intensity at 502 nm showed a linear response to the concentration of HOCl from 3.0 × 10^−7^ to 1.0 × 10^−5^ mol·L^−1^. The detection limit was estimated to be 1.5 × 10^−7^ mol·L^−1^ for HOCl. The fluorescent probe showed fast response and outstanding selectivity toward HOCl. It owned good biocompatibility and had also been successfully applied in the confocal imaging of exogenous and endogenous HOCl in living cells.

## 1. Introduction

Hypochlorous acid (HOCl), as an important reactive oxygen species (ROS), played a vital role in various physiological and pathological processes [1, 2]. Among various ROS, hypochlorous acid (HOCl), which was produced by H_2_O_2_ and Cl^−^ through myeloperoxidase (MPO) [3, 4], was an effective antibacterial agent in the process of human immune defense [5]. Despite its protective effect on immune systems and human health, irregular levels of HOCl were closely associated with many serious diseases, such as osteoarthritis [6], atherosclerosis [7], kidney disease [8], and neurodegeneration [9]. Thus, monitoring the cellular HOCl concentration was very important for biological research and clinical diagnosis.

Many analytical methods had been utilized to detect HOCl, such as electrochemical analysis [10], mass spectrometry [11], high-performance liquid chromatography [12], ultraviolet-visible spectrophotometry [13], chemiluminescence detection [14], and fluorometry [15]. In these methods, fluorescent assay for HOCl possessed the prominent characteristics of rapidity, high sensitivity, and selectivity, noninvasiveness, and real-time determination [16–18]. Therefore, most fluorescent probes for detecting HOCl had been reported. The design strategy of most HOCl fluorescent probes was based on the specific reaction between the recognition group and HOCl, thereby producing a strong fluorescent product. These HOCl recognition groups included *p*-methoxyphenol [19], *p*-aminophenyl ether [20, 21], thioether [22–24], thioester [25, 26], hydrazide [27–29], rhodamine hydroxamic acid [30], selenide [31, 32], unsaturated C = C [33–35], oxime [36, 37], thiocarbamate [38, 39], and others [40]. These specific reactions could effectively distinguish HOCl from other ROS. However, some of these HOCl fluorescent probes had some limitations for the detection of real samples such as relatively slow response time [27, 40] and comparatively low sensitivity [31, 36]. Thus, it was still desirable that a new HOCl fluorescent probe possessed rapid response time and high sensitivity.

**Scheme 1:**
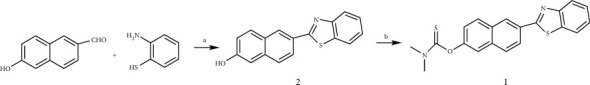
The prepared procedure of probe 1: (a) ethanol, p-toluenesulfonic acid monohydrate, reflux, 12 h 76%; (b) anhydrous dichloromethane, *N, N-*diisopropylethylamine, dimethylaminothioformyl chloride, room temperature, 24 h 70%.

Naphthalene derivatives owing a donor-*π*-acceptor (D-*π*-A) structure had been extensively applied to construct fluorescent probes due to their many advantages such as superior light stability, better chemical stability, and high fluorescence quantum yield. In the past few years, the naphthalene-based fluorescent probes for various substances had been reported including thiophenols [41], HNO [42], H_2_O_2_ [43], cysteine [44], hydrogen polysulfides [45], ONOO^−^ [46], and F^−^ [47]. In this study, a fluorescent probe 1 for HOCl was designed and prepared based on a naphthalene derivative (Scheme 1). *N*, *N*-Dimethylthiocarbamate was applied as a sensing unit for HOCl. Studied results demonstrated the probe possessed excellent sensitivity, a wide pH range, superior selectivity, and fast response time. Furthermore, the probe displayed almost no cell cytotoxicity and had been effectively utilized in the confocal imaging of exogenous and endogenous HOCl in living cells.

## 2. Experimental

### 2.1. Materials and Instruments

Dimethylaminothioformyl chloride and *N*, *N*-diisopropylethylamine were bought from Tianjin Heowns Biochemical Technology Company. *p*-Toluenesulfonic acid monohydrate was purchased from China National Pharmaceutical Group Chemical Reagent Company. 6-Hydroxy-2-naphthaldehyde and 2-aminothiophenol were obtained from Saen Chemical Technology (Shanghai) Company. Sodium hypochlorite was purchased from Tianjin Fuyu Fine Chemical Company. Hydrogen peroxide (H_2_O_2_) and tertbutyl hydroperoxide (TBHP) were obtained from 30% to 70% aqueous solutions, respectively. Hydroxyl radical (·OH) and tert-butoxy radical (O^*t*^Bu) were produced by reaction of 1 mM Fe^2+^ with 200 *µ*M H_2_O_2_ or 200 *µ*M TBHP, respectively. Superoxide (O_2_^−^) was generated from potassium superoxide (KO_2_) solid diluted in dimethyl sulfoxide (DMSO). Singlet oxygen (^1^O_2_) was yielded by the reaction of 1 mM ClO^−^ with 200 *μ*M H_2_O_2_. Peroxynitrite (ONOO^−^) solution was prepared by the reaction of H_2_O_2_ and NaNO_2_, and its concentration was assessed from absorption at *λ* = 302 nm (*ɛ* = 1670 L·mol^−1^·cm^−1^) [40]. Unless otherwise specified, all other chemical reagents were of analytical purity, obtained from commercial suppliers and used without further purification. Silica gel used for thin layer chromatography is 60 F254, and the silica gel used in column chromatography is 200–300 mesh, both of which were obtained from the Qingdao Ocean Chemicals (Qingdao, China). Water was purified by the SZ-93 automatic double pure water distiller (Shanghai Yarong Biochemical Instrument Factory) and used for the preparation of all aqueous solutions. HOCl was standardized at pH 12 (*ɛ*_292 nm_ = 350 L·mol^−1^·cm^−1^) [40].

The NMR spectrum was measured with a Bruker DRX-500 NMR spectrometer using tetramethylsilane (TMS) as the internal standard. The mass spectrum was obtained on an Agilent 6420 triple quadrupole LC/MS high resolution mass spectrometer. Fluorescence tests were performed on a Hitachi F-7000 fluorescence spectrophotometer equipped with a 1 cm quartz absorption cell (Tokyo, Japan). The UV-visible absorption spectrum was measured by an Evolution 260 Bio UV-Vis spectrophotometer with a 1 cm quartz absorption cell. The pH values were measured by the Mettler Toledo pH meter. Fluorescence imaging of living cells was recorded by an Olympus FV-1200 single photon laser confocal microscope. Data processing was mainly obtained in SigmaPlot software. The data obtained by fluorescence spectrophotometry and UV-visible spectrophotometry were measured in 0.01 mol·L^−1^ PBS buffer (DMF/Water = 2:8, V/V, pH = 7.40). Except the fluorescence data recorded by time scanning, all other fluorescence and absorption data were measured at 3 min after adding HOCl at room temperature.

### 2.2. Syntheses

The synthetic route for fluorescence probe 1 is illustrated in Scheme 1.

#### 2.2.1. Synthesis of Compound 2

2-Aminothiophenol (0.69 g, 5.5 mmol) was dissolved in 5 mL ethanol and then added dropwisely to a 35 mL ethanol solution containing 6-hydroxy-2-naphthaldehyde (0.86 g, 5 mmol) and *p*-toluenesulfonic acid monohydrate (1.90 g, 10 mmol) at room temperature. Then, the reaction mixture was heated to reflux for 12 hours, and the solvent was evaporated under reduced pressure to obtain a crude product. The crude product was subjected to column chromatography with petroleum ether/ethyl acetate (4:1, V/V) as the eluent to obtain compound 2 (1.05 g, 76%) as a yellow solid. ^1^H NMR (500 MHz, DMSO-*d*_*6*_), *δ* (ppm): 10.11 (1H, s), 8.54 (1H, s), 8.14 (1H, *d*, *J* = 7.9 Hz), 8.08 (1H, dd, *J* = 8.6 Hz, *J* = 1.6 Hz), 8.05 (1H, *d*, *J* = 8.0 Hz), 8.00 (1H, *d*, *J* = 8.8 Hz), 7.84 (1H, *d*, *J* = 8.6 Hz), 7.55–7.52 (1H, m), 7.46–7.43 (1H, m), 7.20–7.16 (2H, m). ^13^C NMR (125 MHz, DMSO-*d*_6_), *δ* (ppm):168.19, 157.63, 154.18, 136.63, 134.82, 131.20, 127.83, 127.78, 127.74, 127.57, 127.06, 125.75, 124.65, 123.08, 122.73, 120.26, 109.40. MS (ESI) m/z: 278.0638 (M + H)^+^.

#### 2.2.2. Synthesis of Probe 1

Under nitrogen flow, compound 2 (0.28 g, 1 mmol) was dissolved in 20 mL mixture solvent (anhydrous CH_2_Cl_2_: anhydrous DMF = 4:1, V/V). Then, 0.45 mL *N*, *N*-diisopropylethylamine (DIPEA) was added to the above solution. Next, *N*, *N*-dimethylaminothioformyl chloride (0.61 g, 5 mmol) was slowly added to the above mixed solution in batches. The reaction mixture was stirred at room temperature. Then, the solvent was evaporated under reduced pressure, and the crude product was purified by column chromatography using dichloromethane/methanol (50:1, V/V) as the eluent to obtain probe 1 (0.25 g, 70%). ^1^H NMR (500 MHz, CDCl_3_), *δ* (ppm): 8.58 (1H, s), 8.21 (1H, dd, *J* = 8.6 Hz, 1.1 Hz), 8.11 (1H, *d*, *J* = 8.1 Hz), 7.97 (1H, *d*, *J* = 8.8 Hz), 7.93–7.89 (2H, m), 7.53–7.49 (2H, m), 7.41–7.38 (1H, m), 7.31 (1H, dd, *J* = 8.8 Hz, 2.1 Hz), 3.48 (3H, s), 3.40 (3H, s). ^13^C NMR (125 MHz, CDCl_3_), *δ* (ppm): 187.52, 168.05, 153.76, 152.89, 135.03, 134.86, 131.24, 130.64, 129.91, 128.66, 127.58, 126.54, 125.40, 125.07, 123.72, 123.12, 121.66, 119.68, 43.32, 38.85. MS (ESI) m/z: 365.0790 (M + H)^+^, 387.0610 (M + Na)^+^.

### 2.3. Cytotoxicity Assay

To assess the cytotoxicity of the probe, 3-(4, 5-dimethylthiazole-2)-2, 5-diphenyltetrazolium bromide (MTT) assay was carried out. First, PC-12 cells were hatched in 1640 medium supplemented with 10% fetal bovine serum, 100 units mL^−1^ penicillin, and 100 *μ*g mL^−1^ streptomycin. The PC-12 cells in the logarithmic growth phase were planted in a 96-well plate with about 1 × 10^4^ cells in each well, and the total volume of each well was 100 *μ*L. The cells were cultured in an incubator containing 5% CO_2_ for 24 h. The medium was deserted and cleaned with Dulbecco's phosphate buffered saline (DPBS) for three times. Next, the fresh media containing probe 1 (0, 2, 4, 8, and 16 *μ*M) and the purified product of compound 1 with HOCl (0, 2, 4, 8, and 16 *μ*M) were introduced into the wells, respectively, and incubated for 24 h. Subsequently, 10 *μ*L MTT (5 mg·mL^−1^) was added to per well, and the PC-12 cells were fostered for 4 h allowing the formation of formazan. Finally, the above loading media was deserted, and 150 *μ*L DMSO was added to the wells. The 96-well plates were shaken for 10 min, and the absorbance at 490 nm was measured utilizing a microplate reader (Synergy 2, BioTek Instruments Inc.). The survival rate was assessed referring to *A*/*A*_0_ × 100% (*A* and *A*_0_ denote the absorbance of the experimental group and control group, respectively). Similarly, MTT assay of the probe for RAW 264.7 cells was performed.

### 2.4. Confocal Imaging in Living Cells

The PC-12 cells and RAW 264.7 cells were incubated in laser confocal culture dishes at 37°C for 24 h to allow good cell growth and then cleaned three times with DPBS. In the experiment of imaging exogenous HOCl, PC-12 cells were cultured in a medium containing 5.0 *μ*M fluorescent probe 1 at 37°C for 30 min and then washed with DPBS for three times and imaged. In the control experiment imaging exogenous HOCl, PC-12 cells were cultured in the medium containing 5.0 *μ*M probe for 30 min at 37°C, and the supernatant was sucked out. After cleaning with DPBS, the complete culture medium containing 10 *μ*M HOCl was added, incubated for another 30 min, and imaged. The PC-12 cells were washed with DPBS for three times for imaging. In the experiment of imaging endogenous HOCl, RAW 264.7 cells were cultured in a medium containing 5.0 *μ*M fluorescent probe 1 at 37°C for 30 min and then washed with DPBS for three times and imaged. In the control experiment imaging endogenous HOCl, RAW 264.7 cells were stimulated with PMA (1.5 *μ*g/mL) for 30 min and then incubated with 5.0 *µ*M probe 1 for 30 min and imaged. Next, RAW 264.7 cells were stimulated with 1.5 *μ*g/mL PMA for 30 min, cultured with 5 *μ*g/mL taurine, incubated with 5.0 *µ*M probe 1 for 30 min, and imaged. The fluorescence imaging of cells was acquired on the Olympus FV1200-MPE single photon confocal inverted microscope with a 40× objective lens.

## 3. Results and Discussion

### 3.1. Spectroscopic Analytical Characteristic of Probe 1 for HOCl

**Figure 1:**
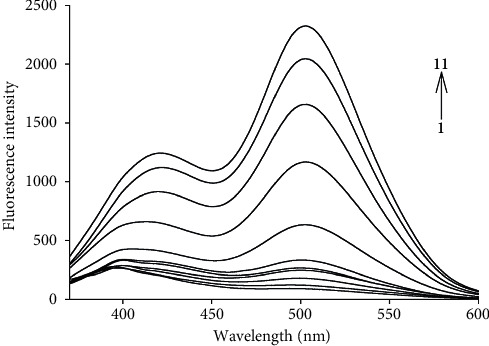
Fluorescence spectra of probe 1 after adding various concentrations of HOCl: 0, 0.30, 0.50, 0.70, 0.90, 1.0, 3.0, 5.0, 7.0, 9.0, 10 *μ*M from 1 to 11 (*λ*ex = 350 nm).

In order to investigate the fluorescence recognizing properties of the probe for HOCl, the emission spectra in the absence and presence of HOCl were recorded in 0.01 mol·L^−1^ PBS buffer (DMF/H_2_O = 2:8, V/V, pH = 7.40) (Figure 1). As shown in Figure 1, before adding HOCl, the free probe displayed weak fluorescence at 502 nm because the internal charge transfer (ICT) process was inhibited. Upon the addition of gradually increased concentration of HOCl, the probe emitted a gradually increased enhancement in fluorescence at 502 nm because the ICT process was resumed. In the presence of 10 *μ*M HOCl, about 25 times fluorescence enhancement at 502 nm was observed. The above findings established the basis for the determination of HOCl concentration with probe 1 developed in the present study. We also investigated the absorption spectra of probe 1 in the absence and the presence of HOCl (Figure S1). As shown in Figure S1, when HOCl was absent, free probe 1 exhibited a maximum absorption peak at 323 nm. After adding HOCl, a major absorption band at 333 nm was observed.

### 3.2. Rule of Operation and the Base of Quantitative Assay

**Figure 2:**
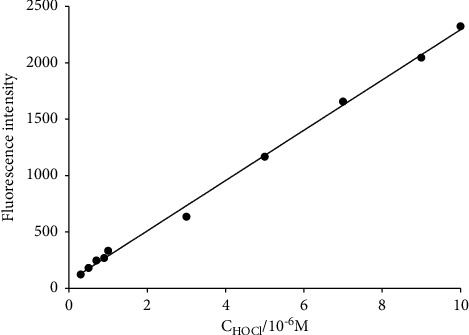
Plot of the fluorescence intensity of probe 1 (5.0 *μ*M) as a function of the concentration of HOCl from 0.30 to 10 *μ*M (*λ*em = 502 nm).

In order to study the linear relationship between the fluorescence intensity of probe 1 and the concentration of HOCl, different concentrations of HOCl were added to the probe. The excitation wavelength of probe was 350 nm. When the concentration of HOCl was in the range of 3.0 × 10^−7^ mol·L^−1^ to 1.0 × 10^−5^ mol·L^−1^, the fluorescence intensity of probe had a linear relationship with the concentration of HOCl (Figure 2). The linear regression equation was *F* = 63.5503 + 223.0080 × 10^6^ × *C* (*r* = 0.9988), where *F* denotes the measured fluorescence intensity, *C* represents the concentration of HOCl, and *r* is the linear correlation coefficient. The detection limit was calculated by three times standard deviation of blank solution [48, 49]. The detection limit was found to be 1.5 × 10^−7^ mol·L^−1^ for the fluorescent probe. Compared with the detection limit of 5.86 × 10^−7^ mol·L^−1^ in reference [31] and the detection limit of 5.8 × 10^−7^ mol·L^−1^ in reference [36], the fluorescent probe in this work displayed a higher sensitivity. The investigated findings illustrated that the probe could be applied as a high sensitive fluorescence probe for quantitative determination of HOCl.

### 3.3. Time-Dependent Response of Probe 1 toward HOCl

**Figure 3:**
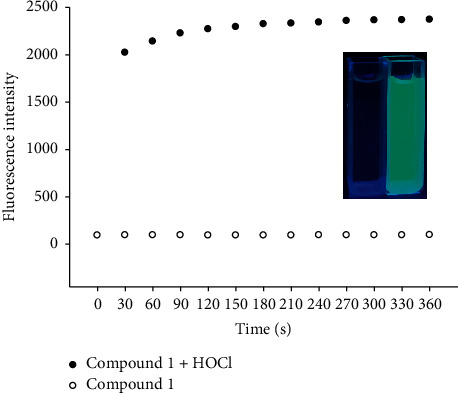
Time course of fluorescence intensity of probe 1 (5.0 *µ*M) in the absence (filled circles) and presence of 10 *µ*M HOCl (clear circles). The inset shows the visual fluorescence color of probe 1 (5.0 *µ*M) before (left) and after (right) incubation with10 *µ*M HOCl for 3 min (UV lamp, 365 nm).

We studied the time response of probe for HOCl by recording the fluorescence intensity of the probe changes with time before and after adding 10 *µ*M HOCl (Figure 3). As shown in Figure 3, in the absence of HOCl, there was no change in the fluorescence intensity of the probe with time. When HOCl was added, the maximum value reached at about 3 min, indicating that the probe responded extremely fast. In the present study, an assay time of 3 min was applied as the measurement condition.

### 3.4. Effect of pH

**Figure 4:**
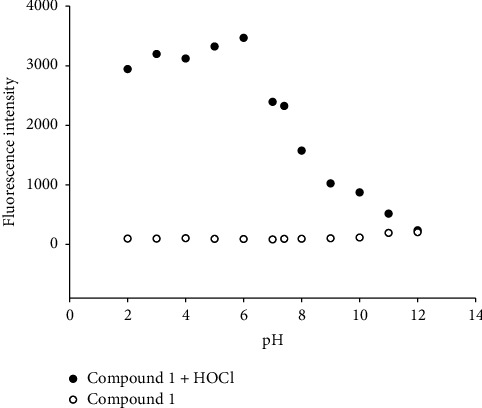
Influence of pH on fluorescence intensity of 5.0 *µ*M probe in the absence (clear circles) and presence of 10 *µ*M HOCl (filled circles).

In order to investigate whether the probe had the ability to respond to HOCl under physiological conditions, the effect of pH on its fluorescence performance was studied. The changes of probe 1 (5 *µ*M) without and with HOCl (10 *µ*M) at different pH values were recorded (Figure 4). As shown in Figure 4, the probe exhibited a high sensitivity for HOCl in the pH range of 2.00–11.00. The above studied results demonstrated that the probe could work in a larger pH range and could be applied for HOCl determination in the biological system.

### 3.5. Selectivity

**Figure 5:**
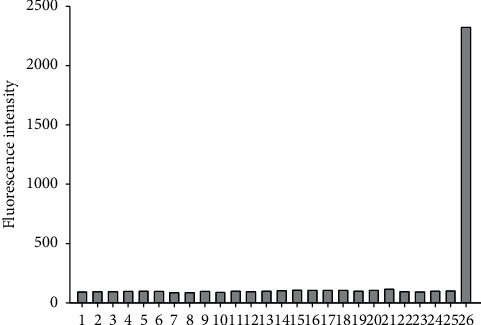
Fluorescence response of the probe (5.0 *µ*M) toward various analytes in 0.01 mol·L^−1^ PBS buffer (DMF/water = 2:8, V/V, pH = 7.40). The fluorescence intensity plotted versus substances: (1) blank; (2) 0.5 mM K^+^; (3) 0.5 mM Na^+^; (4) 0.5 mM Ca^2+^; (5) 0.5 mM Mg^2+^; (6) 0.5 mM Zn^2+^; (7) 0.5 mM Fe^2+^; (8) 10 *μ*M Fe^3+^; (9) 0.5 mM Al^3+^; (10) 0.5 mM NO_2_^−^; (11) 0.5 mM NO_3_^−^; (12) 0.5 mM Cu^2+^; (13) 0.5 mM Pb^2+^; (14) 10 *μ*M Ag^+^; (15) 0.1 mM Cys; (16) 0.1 mM GSH; (17) 0.1 mM Hcy; (18) 0.5 mM H_2_O_2_; (19) 0.5 mM TBHP; (20) 0.5 mM O_2_^−^; (21) 10 *μ*M ^1^O_2_; (22) 10 *μ*M OH; (23) 10 *μ*M·O^*t*^Bu; (24) 0.1 mM ascorbic acid; (25) 10 *μ*M ONOO^−^; (26) 10 *μ*M HOCl.

The selectivity of the fluorescent probe determined its availability in actual samples, so we conducted a selective investigation on probe (Figure 5). As shown in Figure 5, when the pH was 7.40, we studied the fluorescence intensity of probe for HOCl and other related substances including reactive oxygen species (ROS), reactive nitrogen species (RNS), and metal ions. When 10 *μ*M HOCl was added, the fluorescence intensity of the probe increased significantly, and there was no obvious response to other substances. These investigated findings demonstrated that the probe 1 had high selectivity for HOCl.

### 3.6. Proposed Sensing Mechanism

**Scheme 2:**
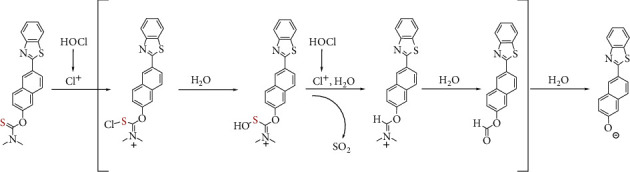
Proposed reaction mechanism of probe 1 with HOCl.

Referring to previous literature [38, 39, 50–52], we evaluated that the production of intermediate immonium was first induced by Cl^+^ from the decomposition of HOCl, and the sequent attack of Cl^+^ resulted in unstable formate ester after the hydrolysis; as a result, compound 2 was yielded through the sequent hydrolysis (Scheme 2). In order to disclose the sensing mechanism of the probe for HOCl, we investigated the reaction mixture of probe 1 with HOCl by the high resolving mass spectrum (HRMS) (Figure S2). As shown in Figure S2, a novel mass peak at m/z 276.0493 appeared, which demonstrated the yield of compound 2 (calcd m/z 276.0489 [M-H]^−^). The above investigated results confirmed the proposed response mechanism of the probe toward HOCl in Scheme 2.

### 3.7. Cytotoxicity Assays and Confocal Imaging in Living Cells

The cytotoxicity was an important factor to estimate the performance of the probe. MTT assay was applied to measure the cytotoxicity. We used the MTT method to determine the cytotoxicity of probe 1 and compound 2 to PC-12 cells and RAW 264.7 cells at different concentrations (0, 2, 4, 8, and 16 *µ*M) (Figure S3). As shown in Figure S3, when probe and compound 2 were present, the cell survival rate reaches more than 85%, indicating that probe and compound 2 are almost nontoxic to PC-12 cells and RAW 264.7 cells.

**Figure 6:**
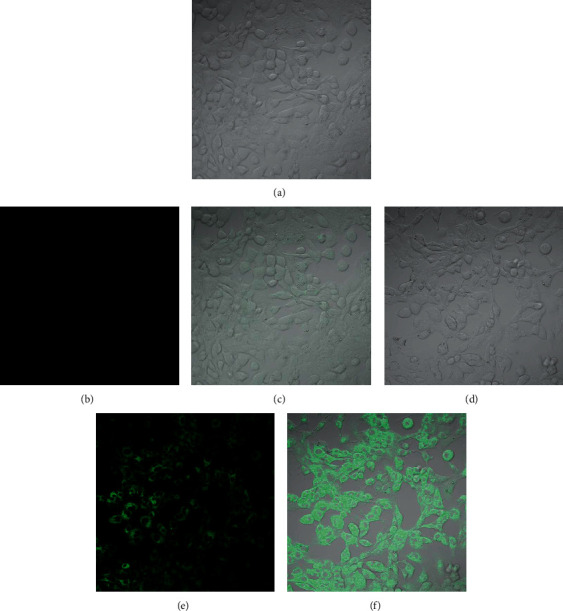
Laser confocal fluorescence imaging of fluorescent probe for HOCl in PC-12: (a) bright field image after incubating PC-12 cells with 5.0 *µ*M probe 1 for 30 min; (b) fluorescence image from green channel of image (a); (c) the overlay of (a) and (b); (d) bright field image of PC-12 cells incubated with 5.0 *µ*M probe 1 for 30 min and then incubated with 10 *μ*M HOCl for 30 min; (e) fluorescence image from green channel of image (d); (f) the overlay of (d) and (e).

**Figure 7:**
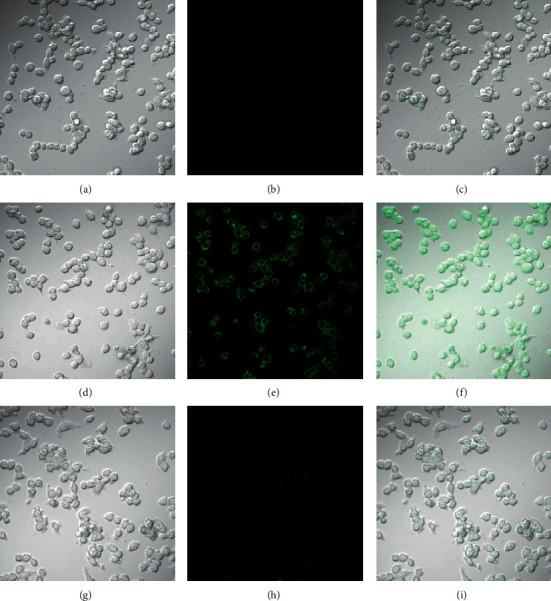
Laser confocal fluorescence imaging of fluorescent probe for HOCl in RAW 264.7 cells: (a) bright field image after incubating RAW 264.7 cells with 5.0 *µ*M probe 1 for 30 min; (b) fluorescence image from green channel of image (a); (c) the overlay of (a) and (b); (d) bright field image of RAW 264.7 cells stimulated with PMA (1.5 *μ*g/mL) for 30 min and then incubated with 5.0 *µ*M probe 1 for 30 min; (e) fluorescence image from green channel of image (d); (f) the overlay of (d) and (e); (g) bright field image of RAW 264.7 cells stimulated with 1.5 *μ*g/mL PMA for 30 min, cultured with 5 *μ*g/mL taurine, and then incubated with 5.0 *µ*M probe 1 for 30 min; (h) fluorescence image from green channel of image (g); (i) the overlay of (g) and (h).

In order to prove the practicality of probe in biological detection, we used fluorescent probe to perform laser confocal fluorescence imaging of HOCl in PC-12 cells and RAW 264.7 cells. PC-12 cells were seeded in a 35 mm laser confocal culture dish and cultured for 24 hours. After incubating for 30 min in a medium containing 5.0 *µ*M probe for fluorescence imaging, it was found that the green channel had almost no fluorescence (Figure 6(b)). At the same time, PC-12 cells were incubated with 5.0 *µ*M probe for 30 min and then incubated with 10 *µ*M HOCl for another 30 min and imaged. As shown in Figure 6(e), the fluorescence of the green channel was much stronger than that without HOCl. Next, we further assessed the capability of the probe 1 for imaging endogenous HOCl. The trial was conducted in RAW 264.7 cells because the cells could generate high levels of HOCl after being stimulated by phorbol-12-myristate-13-acetate (PMA) [51, 52]. The RAW 264.7 cells were hatched with 5.0 *µ*M probe, and weak fluorescence was observed in green channel (Figure 7(b)). When the cells were treated with 1.5 *μ*g/mL PMA for 30 min and then hatched with 5.0 *µ*M probe for 30 min, a remarkable fluorescence increase in RAW 264.7 cells was found (Figure 7(e)). However, after the cells stimulated with PMA were treated with taurine, which was an amino acid that could capture HOCl [52], and then incubated with 5.0 *µ*M probe for 30 min, much weaker fluorescence was obtained (Figure 7(h)). Meanwhile, it also illustrated that the observed intracellular fluorescence enhancement was induced by the endogenous HOCl and no other ROS. These experimental results demonstrated that probe could be utilized for laser confocal imaging of exogenous and endogenous HOCl in living cells.

## 4. Conclusions

On the whole, we constructed and prepared a novel naphthalene-based fluorescent probe for the determination of hypochlorous acid. *N*, *N*-Dimethylthiocarbamate was chosen as the selective sensing unit for HOCl. When HOCl was absent, the reported probe displayed weak fluorescence. In the presence of HOCl, the probe exhibited green fluorescence. The probe had a short response time, a wide working pH range, and the high selectivity and specificity for hypochlorous acid. At the same time, the probe had good biocompatibility and is suitable for the detection of hypochlorous acid. In addition, confocal fluorescence imaging of living cells showed that the probe could be utilized to image exogenous and endogenous HOCl in living cells.

## Data Availability

The data used to support the findings of this study are included within the article and Supplementary Materials.

## Supplementary Materials

Supplementary Materials Figure S1. Absorption spectra of fluorescent probe 1 (50 *µ*M) and the reaction product of fluorescent probe 1 (50 *µ*M) with HOCl (200 *µ*M). The solid line (—) and dotted line (···) represent fluorescent probe 1 and the reaction product of fluorescent probe 1 with HOCl, respectively. Figure S2. HRMS spectrum of the reaction mixture of probe 1 with HOCl. Figure S3. MTT assay of PC-12 cells in the presence of different concentrations of probe 1 (A) and compound 2 (B) for 24 h at 37°C, respectively; MTT assay of RAW 264.7 cells in the presence of different concentrations of compound 1 (C) and compound 2 (D) for 24 h at 37°C, respectively. Figure S4. ^1^H NMR spectrum of compound 2 in DMSO-*d*_*6*_. Figure S5. ^13^C NMR spectrum of compound 2 in DMSO-*d*_*6*_. Figure S6. MS spectrum of compound 2. Figure S7. ^1^H NMR spectrum of compound 1 in CDCl_3_. Figure S8. ^13^C NMR spectrum of compound 1 in CDCl_3_. Figure S9. MS spectrum of compound 1.
