# Supplementary material for: A Highly Selective Fluorescent Probe for Hypochlorous Acid in Living Cells Based on a Naphthalene Derivative
Source: Int J Anal Chem. 2022 Feb 14;2022:7649230. doi: 10.1155/2022/7649230 (PMC8860552; doi:10.1155/2022/7649230)
Supplement: Supplementary Materials — Figure S1. Absorption spectra of fluorescent probe 1 (50 µM) and the reaction product of fluorescent probe 1 (50 µM) with HOCl (200 µM). The solid line (—) and dotted line (···) represent fluorescent probe 1 and the reaction product of fluorescent probe 1 with HOCl, respectively. Figure S2. HRMS spectrum of the reaction mixture of probe 1 with HOCl. Figure S3. MTT assay of PC-12 cells in the presence of different concentrations of probe 1 (A) and compound 2 (B) for 24 h at 37°C, respectively; MTT assay of RAW 264.7 cells in the presence of different concentrations of compound 1 (C) and compound 2 (D) for 24 h at 37°C, respectively. Figure S4. 1H NMR spectrum of compound 2 in DMSO-d6. Figure S5. 13C NMR spectrum of compound 2 in DMSO-d6. Figure S6. MS spectrum of compound 2. Figure S7. 1H NMR spectrum of compound 1 in CDCl3. Figure S8. 13C NMR spectrum of compound 1 in CDCl3. Figure S9. MS spectrum of compound 1. [file 7649230.f1.zip › Supplementary Materials/7649230.f1.docx]

**Supporting Information**

**A highly selective fluorescent probe for hypochlorous acid in living cells based on a naphthalene derivative**

Jingguo Sun ^a^, Junhong Xu ^b^, Qiujuan Ma ^* a^, Guojiang Mao^*^ ^c^, Nannan Zhu ^a^, Meiju Tian ^a^, Linke Li ^a^, Shuzhen Liu ^a^

^a^ *School of Pharmacy, Henan University of Chinese Medicine, Zhengzhou 450046, PR China*

^b^ *Department of Dynamical Engineering, North China University of Water Resources and Electric Power, Zhengzhou 450011, PR China*

^c^ *Henan Key Laboratory of Organic Functional Molecule and Drug Innovation, Collaborative Innovation Center of Henan Province for Green Manufacturing of Fine Chemicals, Key Laboratory of Green Chemical Media and Reactions, Ministry of Education, School of Chemistry and Chemical Engineering, Henan Normal University, Xinxiang, 453007, PR China*

*Corresponding author, E-mail: maqiujuan104@126.com (Q. J. Ma); [maoguojiang@htu.edu.cn](mailto:maoguojiang@htu.edu.cn) (G. J. Mao); Tel: +86-371-65676656; Fax: +86-371-65680028.

**Table of contents**

**UV-visible absorption spectroscopy .........................................................................** S3

**HRMS spectrum of the reaction mixture of probe 1 with HOCl ...........................** S4

**MTT assay of living cells ..........................................................................................** S5

**NMR and MS data for compounds** ....................................................................... S6


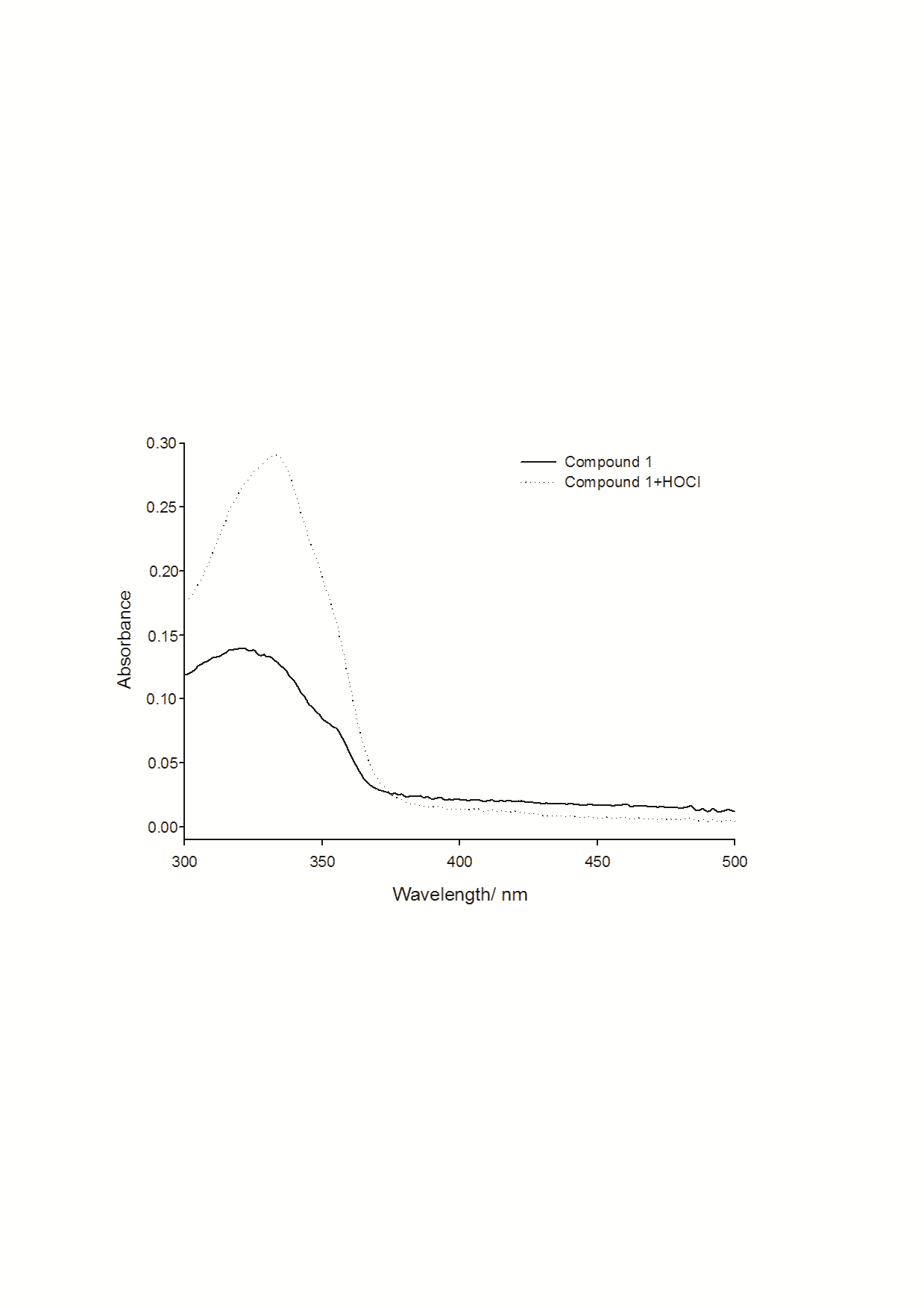


**Figure S1** Absorption spectra of fluorescent probe 1 (50 µM) and the reaction product of fluorescent probe 1 (50 µM) with HOCl (200 µM). The solid line (—) and dotted line (···) represent fluorescent probe **1**, and the reaction product of fluorescent probe **1** with HOCl, respectively.


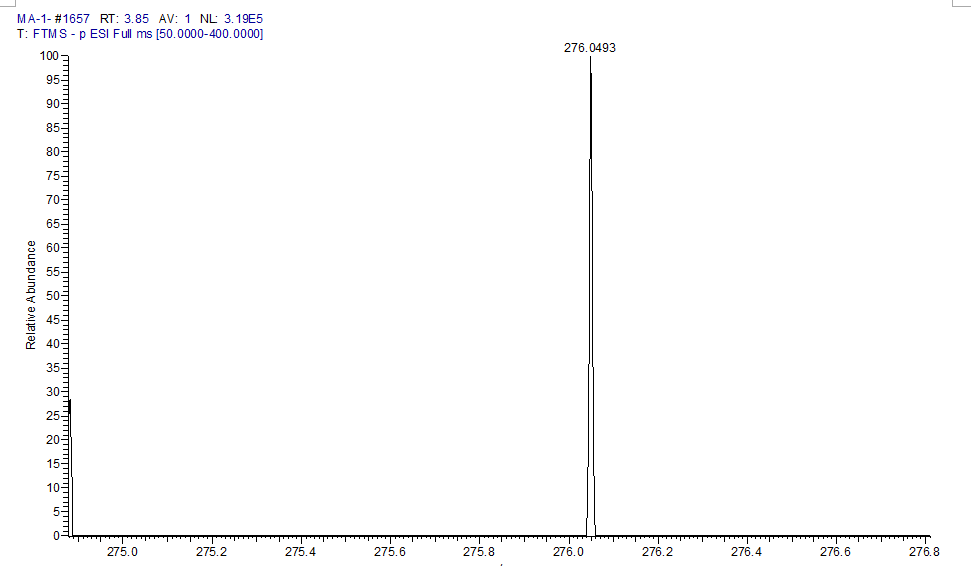


**Figure S2** HRMS spectrum **of** the reaction mixture of probe **1** with HOCl





**Figure S3** MTT assay of PC-12 cells in the presence of different concentrations of probe 1 (A) and compound 2 (B) for 24 h at 37 °C, respectively; MTT assay of RAW 264.7 cells in the presence of different concentrations of compound 1 (C) and compound 2 (D) for 24 h at 37 °C, respectively.

**NMR and MS data for compounds**


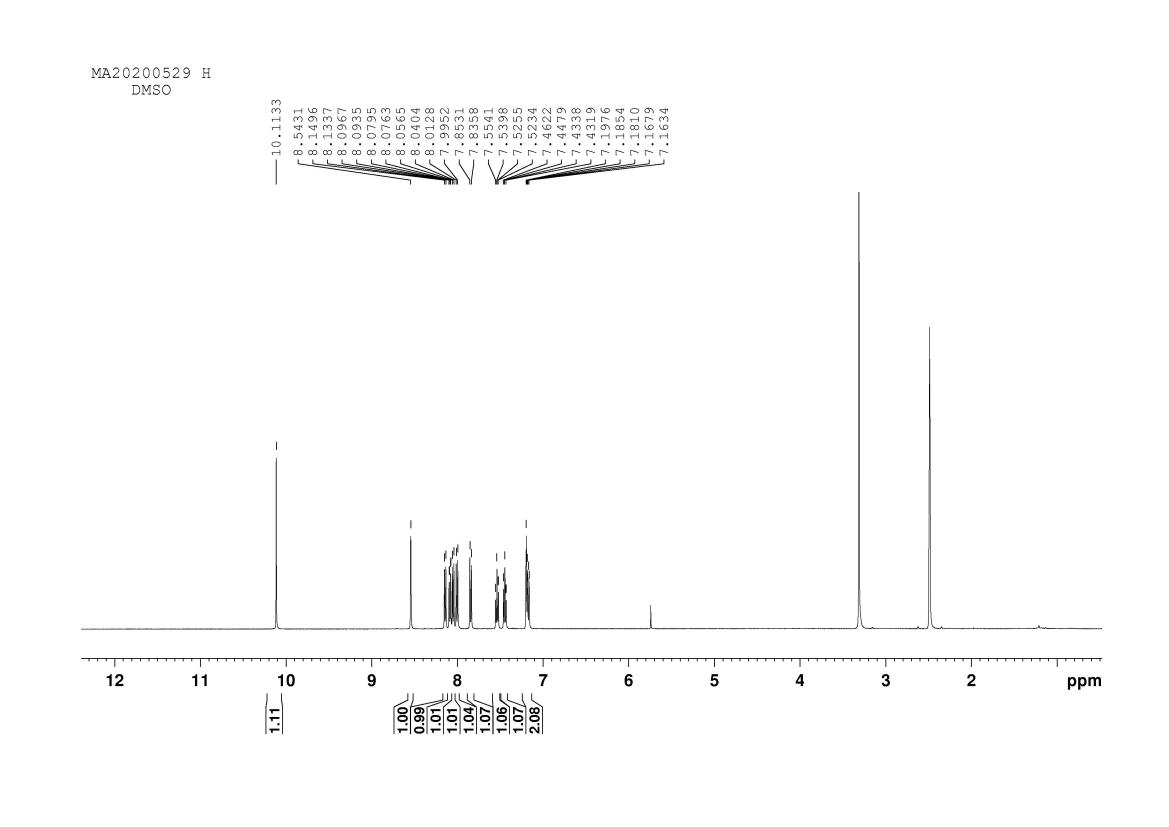


**Figure S4** ^1^H NMR spectrum of compound **2** in DMSO-*d_6_*.

**
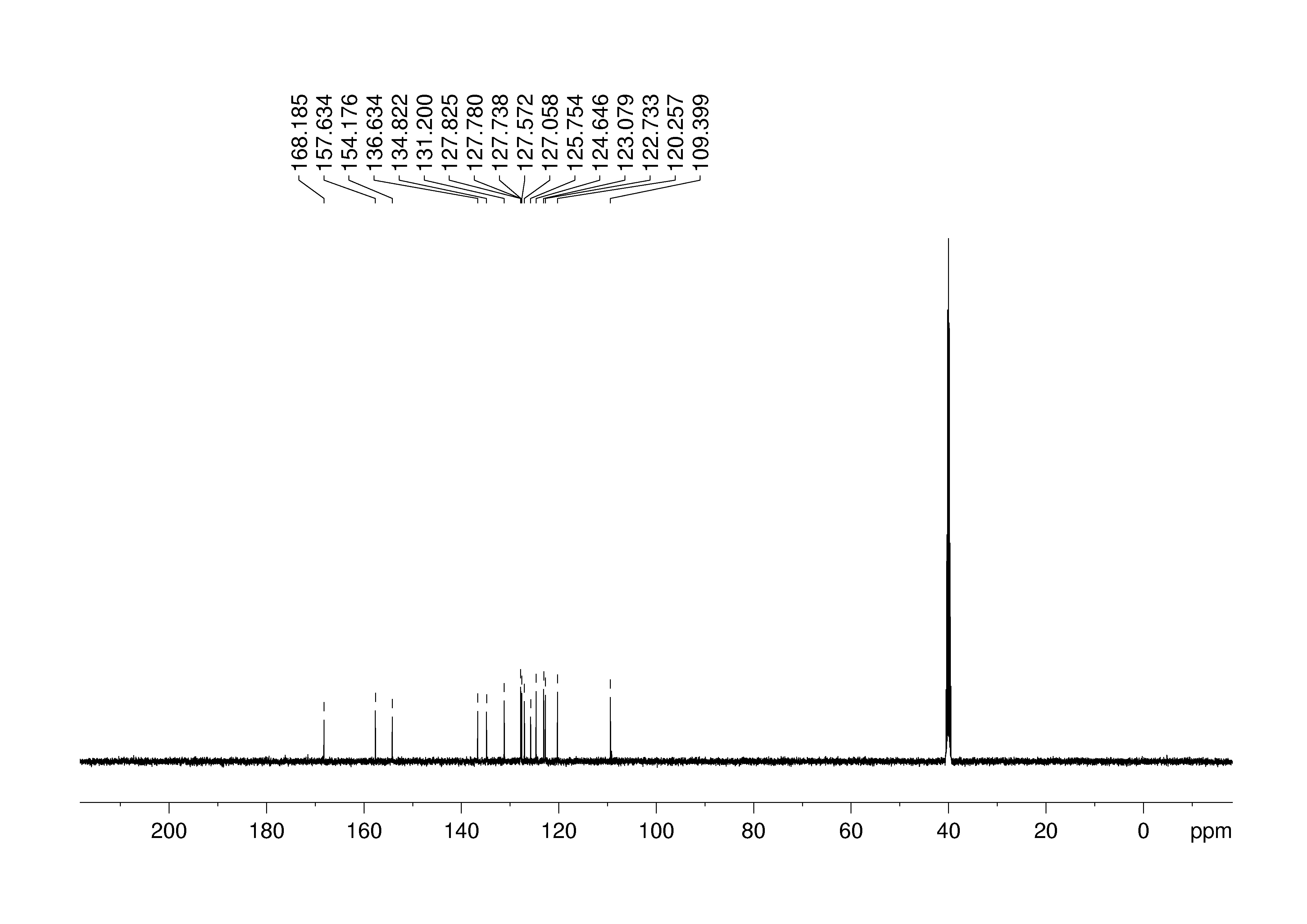
**

**
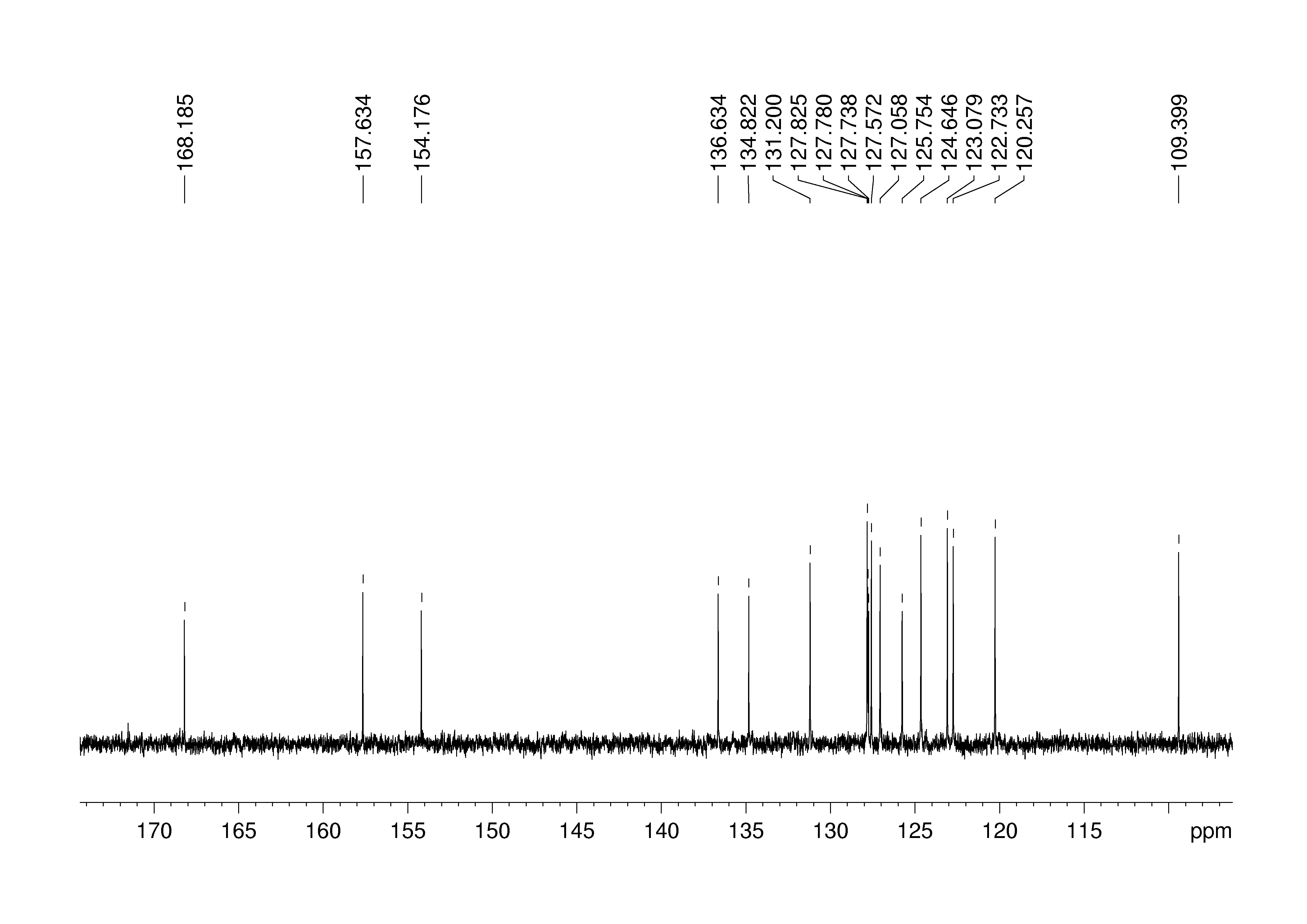
**

**Figure S5** ^13^C NMR spectrum of compound **2** in DMSO-*d_6_*.

**Figure S6** MS spectrum of compound **2**.


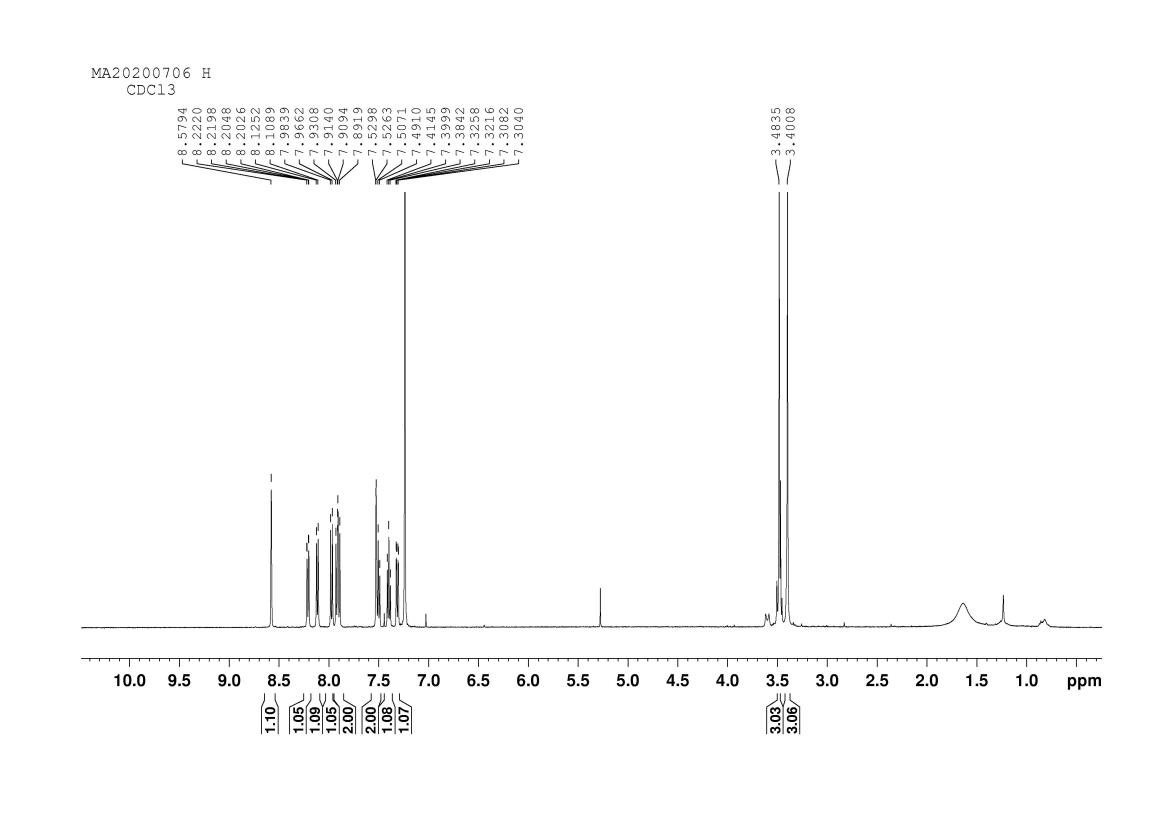


**Figure S7** ^1^H NMR spectrum of compound **1** in CDCl_3_.


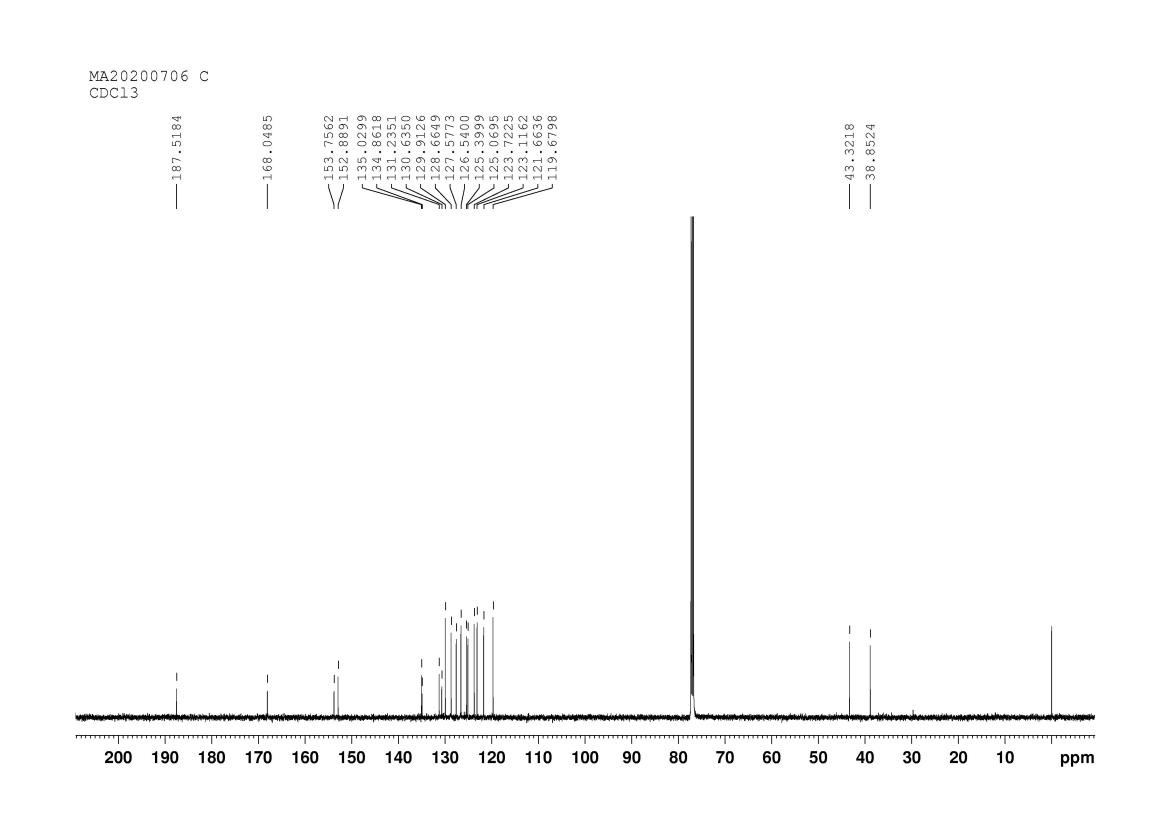


**Figure S8** ^13^C NMR spectrum of compound 1 in CDCl_3_.

**Figure S9** MS spectrum of compound 1.
